# Primary Renal Synovial Sarcoma: A Report of a Rare Case and Management Approach

**DOI:** 10.7759/cureus.100694

**Published:** 2026-01-03

**Authors:** Souvik Mondal, Sandip Kumar Barik, Sambit K Tripathy, Suvendu Purkait, Saroj K Das Majumdar

**Affiliations:** 1 Department of Radiation Oncology, All India Institute of Medical Sciences, Bhubaneswar, Bhubaneswar, IND; 2 Department of Urology, All India Institute of Medical Sciences, Bhubaneswar, Bhubaneswar, IND; 3 Department of Pathology and Lab Medicine, All India Institute of Medical Sciences, Bhubaneswar, Bhubaneswar, IND

**Keywords:** aim chemotherapy, primary renal synovial sarcoma, rare kidney tumour, renal synovial sarcoma, ss18 translocation, translocation study

## Abstract

Primary renal synovial sarcoma (PRSS) is an exceedingly rare malignancy. Due to its rarity, the diagnosis and management of PRSS remain challenging, as there are no standardized treatment guidelines.

We present a case of a 35-year-old male who presented with right flank pain for two months. Contrast-enhanced CT (CECT) revealed a 10 × 11 × 12 cm mass at the upper pole of the right kidney. The patient underwent radical nephrectomy, and histopathological examination suggested synovial sarcoma. To confirm the diagnosis, we performed a translocation study, which identified the SS18 gene translocation at 18q11, a hallmark of synovial sarcoma. Postoperatively, the patient received adjuvant chemotherapy with the AIM (doxorubicin, ifosfamide, and mesna) regimen.

Given the extreme rarity of PRSS, we discuss the diagnostic challenges, molecular characteristics, and treatment approach adopted at our institution, contributing to the limited but growing body of knowledge on this rare entity.

## Introduction

Primary renal synovial sarcoma (PRSS) is a rare presentation of sarcoma of the kidney, with only a few cases reported in the literature, numbering around 200. PRSS presents with an incidence of 1-3% among all renal tumors [1]. Due to the rarity of this disease, standard protocols for diagnosing and treating this rare neoplasm are strongly required. This case study aims to thoroughly discuss the clinical features, morphological and immunochemical findings of PRSS, and the role of current diagnostic and therapeutic management of this aggressive neoplasm [2].

## Case presentation

A 35-year-old male presented with complaints of pain in the right flank for two months, without any history of fever or hematuria. On general examination, there was no pallor, icterus, cyanosis, edema, or clubbing. The patient was a farmer by profession, with no comorbidities, no known addiction, no significant family history of hereditary diseases, and no prior exposure to radiation or cytotoxic drugs. On palpation of the abdomen, a bimanually palpable soft mass was found on the right side, without tenderness. Upon examination of the lymphatic system, there was no lymphadenopathy. Upon system examination, all other systems were within normal limits. On routine investigation, complete blood count and liver function test were normal. Kidney function was deranged, as evidenced by a serum creatinine level of 1.75 mg/dL and a urea level of 30 mg/dL (Table 1). Urine routine and microscopy were within normal limits.

**Table 1:** Values of urea and creatinine before and after surgery with normal reference range.

Test	Before surgery	After surgery	Normal range
Urea (Ur)	30 mg/dL	26 mg/dL	16-42 mg/dL
Creatinine (Cr)	1.75 mg/dL	1.2 mg/dL	0.8-1.2 mg/dL

**Figure 1:**
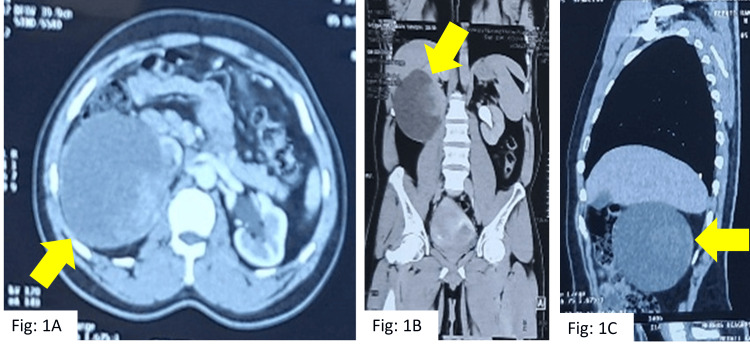
Contrast-enhanced computed tomography (CECT) showing right kidney lower pole cystic renal mass (10 * 11 * 12 cm) with solid component (5 * 5.4 * 4.2 cm). (A) A cystic mass with a solid component is seen in axial CECT (yellow arrow). (B) A large cystic mass can be seen below the liver (arrow), almost occupying the whole kidney. The normal kidney is on the opposite side. (C) Same cystic mass in the sagittal section.

On initial evaluation with USG of the abdomen, an approximately 10 * 9 cm cystic right renal mass was found. On further evaluation with contrast-enhanced computed tomography (CECT), a cystic mass was noted, measuring 10 * 11 * 12 cm, arising from the cortex of the lower pole of the right kidney, with a solid nodule measuring 5.4 * 5 * 4.2 cm (Figure 1). Initially, it was diagnosed as a renal cyst of Bosniak type IV [3]. A 3D laparoscopic right radical nephrectomy was done under general anesthesia in the urosurgery department. For the procedure, standard laparoscopic port placement was done. Following mobilization of the right colon and Kocherization of the duodenum, the renal vein and arteries were identified, clipped, and cut; the right radical nephrectomy was then completed, and the specimen was retrieved via a right iliac fossa incision.

**Figure 2:**
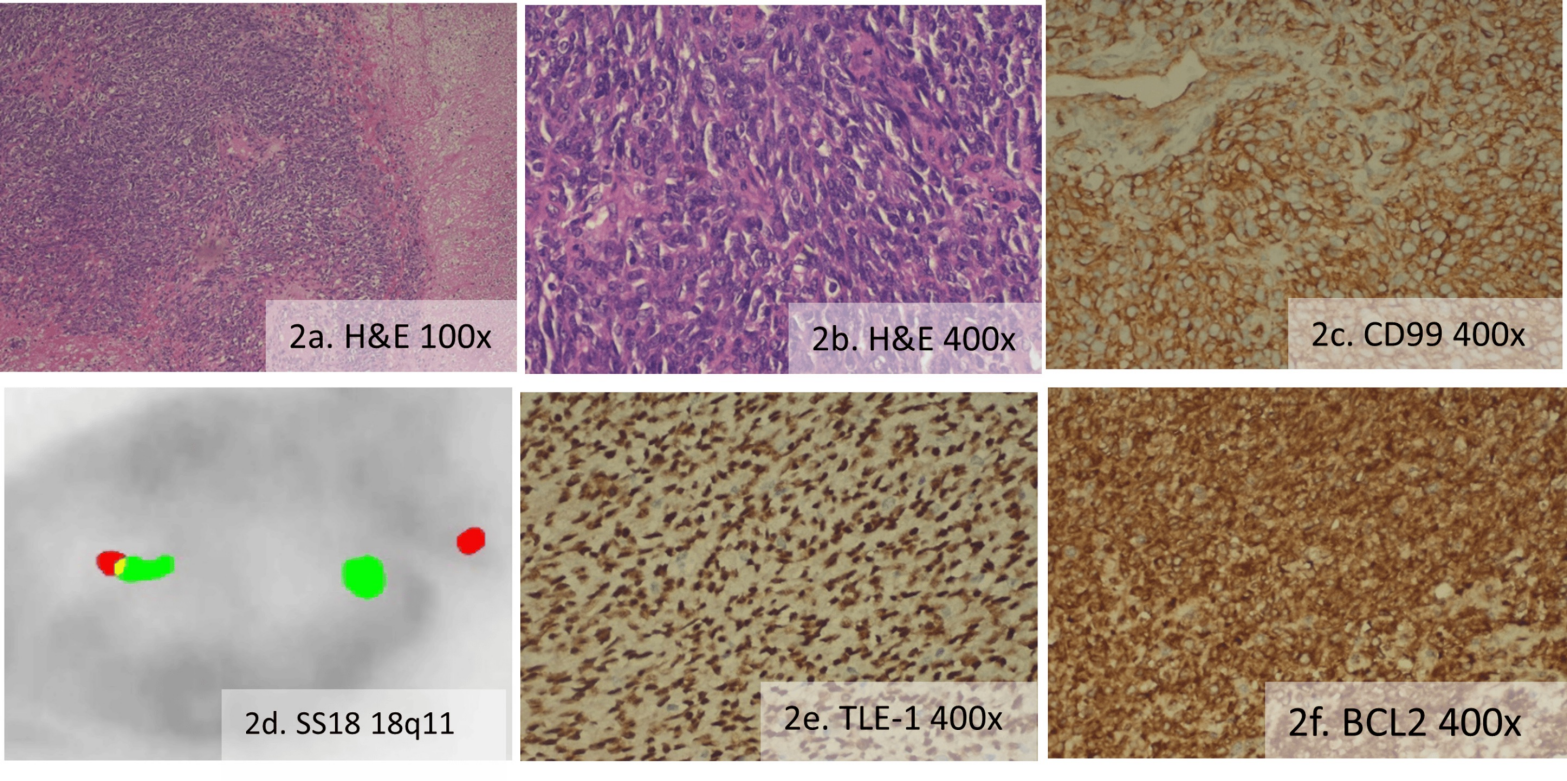
(a) and (b) H&E-stained poorly differentiated synovial sarcoma, (c) IHC for CD99 was positive, (d) translocations of the SS18 gene at 18q11 (5’ SS18: orange; 3’ SS18: green), (e) IHC for TLE-1 was positive, and (f) IHC for BCL2 was positive. H&E: hematoxylin & eosin; IHC: immunohistochemistry.

Histopathology report showed poorly differentiated synovial sarcoma (Figures 2A, 2B) [2], measuring 6.2 * 5.05 * 3.5 cm. All margins were negative, and the specimen showed less than 50% necrosis without invasion of the renal capsule; the grade 3 tumor was arranged in sheets, comprised of spindle-to-oval shaped cells with moderate clear-to-pale eosinophilic cytoplasm, containing spindle and round irregular nuclei with vesicular chromatin and conspicuous nucleoli alongside areas of necrosis and hemorrhage, exhibiting a high mitotic index of 22-23/10 HPF but without any lymphovascular or perineural invasion. The American Joint Committee on Cancer (AJCC) 8th edition TNM (tumor, node, and metastasis) staging was pT2N0M0, group IIIA, strongly positive for CD99 (Figure 2C), BCL2 (Figure 2F), and TLE-1 (Figure 2E), weak focal positive for pan-CK and cyclin-D1, while negative for SMA, CD31, CD34, myogenin, CD10, and GATA3. In view of renal synovial sarcoma, we decided to do a translocation study by fluorescence in situ hybridization (FISH), and it was positive for translocation of the SS18 gene at 18q11 (Figure 2D). With these findings, it was diagnosed as a case of primary renal synovial sarcoma. For metastatic workup, a CECT of the thorax was done, showing no metastatic lesions in the chest.

**Figure 3:**
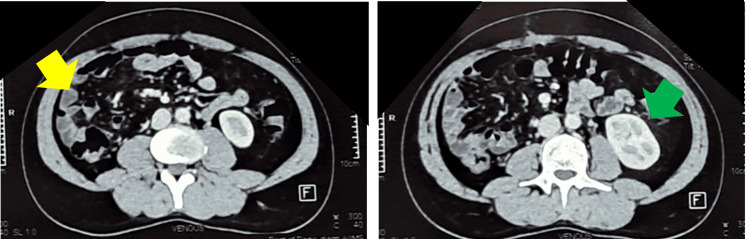
Postoperative contrast-enhanced computed tomography (CECT) showing no residual or recurrent disease. Yellow arrow: postoperative bed; green arrow: normal opposite kidney.

After discussion of the case in the departmental board meeting, adjuvant chemotherapy with the AIM (doxorubicin, ifosfamide, and mesna) regimen was planned, followed by assessment. Post surgery, the patient’s kidney function test improved, and serum creatinine was around the normal range of 1.2 mg/dL (Table 1). So, he was started on the AIM regimen, with Adriamycin 25 mg/m2 on days one to three, ifosfamide 2500 mg/m2 on days one to three, and mesna 1500 mg/m2 on days one to three. Subsequently, the patient received three cycles of chemotherapy and tolerated them well. Post chemotherapy, there was grade 2 neutropenia around days seven to eight, and the patient received prophylactic granulocyte colony-stimulating factor (GM-CSF) pegfilgrastim 6 mg after every cycle of chemotherapy on day four. After three cycles of chemotherapy, assessment was done, and CECT showed no local or distant failure (Figure 3), so we continued with the AIM regimen to complete six cycles of adjuvant therapy.

Post six cycles of chemotherapy, CECT assessment was done, and a report of multiple speculated nodules in bilateral lungs was obtained, with the largest measuring 8 * 6 mm in the left lung, suggestive of metastatic deposit and progressive disease. Now the patient is planned for the second line of chemotherapy with pazopanib 600 mg once daily until disease progression or intolerable toxicities.

## Discussion

Synovial sarcoma (SS) is a rare mesenchymal tumor, constituting approximately 5-10% of all soft tissue sarcomas (STS) [4]. Primary renal sarcomas are exceptionally uncommon, accounting for only 1% of malignant renal neoplasms. This malignancy predominantly affects young adults between 20 and 50 years of age [5]. The most widely accepted hypothesis regarding its origin suggests that it arises from the retrograde differentiation of an undefined mesenchymal cell [6]. Clinicoradiologically differential diagnosis for PRSS includes Wilms’ tumor, mixed epithelial-stromal tumor, sarcomatoid renal cell carcinoma, congenital mesoblastic nephroma, primitive neuroectodermal tumor (PNET), malignant peripheral nerve sheath tumor (MPNST), and hemangiopericytoma [7].

Histologically, SS is classified into biphasic (BSS), monophasic spindle cell (MSSS), and poorly differentiated variants. Among these, poorly differentiated synovial sarcoma (PDSS) comprises approximately 20% of cases and is associated with the poorest prognosis [2]. Immunohistochemically, SS cells typically express vimentin and, in some cases, show focal positivity for Bcl-2 and epithelial membrane antigen (EMA) [8]. Conversely, PDSS are negative for cytokeratin, CD34, protein S-100, and CD117 [4]. More than 90% of SS cases exhibit the t(x;18)(p11;q11) chromosomal translocation, leading to the fusion of the SS18-SSX1 or SS18-SSX2 genes [6]. Histopathological evaluation, performed through hematoxylin and eosin (HE) staining, coupled with immunohistochemistry, plays a crucial role in diagnosis. Cytogenetic and molecular genetic testing further enhance diagnostic precision. Male sex, tumor size more than 5-10 cm, deep fascial invasion, proximal of extremities and truncal location, MSSS, grade 3 with high mitotic rate and marked atypia with spontaneous necrosis more than 25%, and neurovascular invasion are some poor prognostic factors associated with poor survival [9].

The standard treatment for PRSS primarily involves surgical resection, often supplemented by adjuvant chemotherapy. Although preoperative chemotherapy and radiotherapy have been explored, there is insufficient evidence to confirm their superiority in improving clinical outcomes. The role of adjuvant chemotherapy remains a subject of debate, with ongoing registry studies assessing its efficacy. Initial investigations utilizing anthracycline- and ifosfamide-based chemotherapy demonstrated marginal survival benefits, although subsequent large-scale clinical trials failed to validate these findings. PRSS is characterized by a high propensity for local recurrence and distant metastasis, with reported five-year survival rates ranging between 20% and 50% [2].

Targeted therapy represents an evolving approach in PRSS management. Pazopanib, a multi-targeted tyrosine kinase inhibitor (TKI), has demonstrated meaningful clinical activity in metastatic SS as a second-line therapy following anthracycline failure. The drug inhibits vascular endothelial growth factor receptors (VEGFR-1, VEGFR-2, and VEGFR-3), platelet-derived growth factor receptors (PDGFR-α and PDGFR-β), and c-kit, thereby targeting both angiogenic and oncogenic pathways [10]. In the landmark EORTC phase II study, SS showed promising activity with 49% of patients achieving progression-free survival at 12 weeks, and five out of nine partial responses occurring in SS patients [11]. There are ongoing clinical investigations aiming to establish the long-term efficacy of newly approved multiple signaling pathway blocking TKIs, like anlotinib, and their potential role in optimizing treatment outcomes for PRSS patients [12].

## Conclusions

PRSS is an exceedingly rare malignancy, often posing diagnostic and therapeutic challenges due to its histopathological overlap with other renal neoplasms and the absence of standardized treatment guidelines. This case highlights the importance of a multidisciplinary approach in diagnosing and managing PRSS, with molecular studies crucial in confirming the diagnosis. Surgical resection remains the cornerstone of treatment, while adjuvant chemotherapy with an anthracycline- and ifosfamide-based regimen has shown potential benefits in disease control. Despite advances in diagnostic and therapeutic strategies, PRSS continues to exhibit a high recurrence and metastasis rate, leading to a guarded prognosis. Given the rarity of PRSS, continued documentation of cases and collaborative research efforts are essential to enhance our understanding of this aggressive neoplasm and to develop more effective treatment protocols.
